# Effects and mechanisms of a home-based action observation and motor imagery intervention on cognitive function and depression in spinal cord injury: a pilot randomized controlled trial protocol

**DOI:** 10.3389/fneur.2025.1578323

**Published:** 2025-05-16

**Authors:** Yule Hu, Yan Li, Ran Tao, Chun Liang Hsu, Ashley Craig, Chor Yin Lam, Turhan Kahraman, Angela Yee Man Leung

**Affiliations:** ^1^School of Nursing, The Hong Kong Polytechnic University, Hong Kong, Hong Kong SAR, China; ^2^Research Centre for Language, Cognition, and Neuroscience, Department of Chinese and Bilingual Studies, The Hong Kong Polytechnic University, Hong Kong, Hong Kong SAR, China; ^3^The Department of Rehabilitation Sciences, The Hong Kong Polytechnic University, Kowloon, Hong Kong SAR, China; ^4^John Walsh Centre Rehabilitation Research, The Kolling Institute, St Leonards, NSW, Australia; ^5^Faculty of Medicine and Health, Sydney Medical School, The University of Sydney, Sydney, NSW, Australia; ^6^Department of Orthopaedics and Traumatology, School of Clinical Medicine, Li Ka Shing Faculty of Medicine, The University of Hong Kong, Pokfulam, Hong Kong SAR, China; ^7^Department of Physiotherapy and Rehabilitation, Faculty of Health Sciences, Izmir Katip Celebi University, Çigli, Türkiye

**Keywords:** spinal cord injury, cognitive function, depression, motor imagery, rehabilitation, protocol, randomized controlled trial

## Abstract

**Background:**

Cognitive impairment and depression frequently occur after spinal cord injury (SCI) and adversely affect functional independence and quality of life. There is a lack of research in addressing this important area in SCI rehabilitation/care. The home-based Action Observation and Motor Imagery (AOMI), a form of neurorehabilitation, was developed grounded in theoretical foundations and practical evidence, rendering it especially suitable for adults with SCI. This study aims to evaluate the feasibility, acceptability, and preliminary effects of this innovative intervention on SCI adults’ cognitive function and depression, while also exploring the underlying neural mechanisms through multimodal magnetic resonance imaging (MRI).

**Methods:**

This is an assessor-blinded, two-arm pilot randomized controlled trial with repeated measures (pre-, post-intervention, and 1-month follow-up). In total, 46 participants will be randomly assigned to either the intervention group, which will receive an 8-week AOMI intervention combined with basic wheelchair exercises, or the control group, which will watch landscape videos and perform basic wheelchair exercises. The feasibility of study procedures will be assessed by examining the recruitment, dropout, and retention rates. The acceptability of interventions will be evaluated by the adverse events record, satisfaction, and adherence rate. The primary outcomes of intervention effectiveness include global cognitive function and depression; secondary outcomes include neurocognitive domains, multimodal MRI findings, chronic pain, self-efficacy for exercise, and motor imagery ability.

**Conclusion:**

The study findings will preliminarily determine the effects of AOMI in SCI rehabilitation. Multimodal MRI data will elucidate the neuroplastic changes and functional reorganization occurring in the brains of the SCI population following the intervention.

**Clinical trial registration:**

ClinicalTrials.gov, identifier NCT06708026.

## 1 Introduction

Spinal cord injury (SCI) is a kind of neurological disorder that leads to partial or complete loss of sensory and/or motor functions below the injured level (1). Over 20 million individuals worldwide are living with SCI, and this number continues to increase each year (1). Advances in healthcare services have extended life expectancy for SCI survivors, with nearly 50% surviving 40 years post-injury (2). With the rise in life expectancy, survivors have an increased demand for rehabilitation, community reintegration, and returning to school and work. Cognitive function plays an important role in the successful rehabilitation procedure, as it involves intensive learning and the employment of new skills. However, approximately 30%–60% of adults with SCI suffer from cognitive impairment, with 16% exhibiting severe cognitive impairment (3). Cognitive impairment can develop in individuals with SCI of any age and may occur during both acute and chronic phases (4).

Research has shown that SCI survivors who have cognitive impairment are more likely to experience depression after transitioning back into the community compared to those with normal cognitive function, highlighting the strong link between cognitive function and depression in this population (4). Cognitive impairment, especially in attention, could serve as a gateway for negative thoughts and biases, constituting an internal stressor that maintains the depressive state (5). Depression could also affect cognitive function, including information processing and reasoning (5). There are some joint neural mechanisms behind cognitive impairment and depression following SCI. Longitudinal functional magnetic resonance imaging (fMRI) studies have revealed that SCI can lead to a progressive reduction of brain regions, including the cerebellar cortex, medial prefrontal cortex, and anterior cingulate cortex. These areas are essential for emotional processing and cognitive function (6). Chronic neuropathic pain, a frequent secondary complication of SCI, also contributes substantially to the development of both conditions (7). Moreover, pain and reduced physical activity following SCI can impair hippocampal neurons, which play a vital role in cognition and mood regulation (8).

Previous studies mostly focused on the physical or psychological rehabilitation of SCI care. There is a scarcity of randomized controlled trials that particularly address cognitive impairment in this population, let alone studies that jointly address cognitive impairment and depression (9). Action Observation and Motor Imagery (AOMI), a mental simulation process, refers to a person watching a recorded or live demonstration of a movement while simultaneously using imagery to covertly rehearse the same movement without actually executing it (10). AOMI involves the internal simulation of actual movement, thereby inducing neural and autonomic changes similar to those executing real movement (11, 12). As a non-invasive, cost-effective, and accessible therapy, AOMI has been widely applied to physical function improvement, especially for individuals with physical disability or mobility impairment (13). It enables individuals who cannot undergo conventional physical training (e.g., those with severe physical disabilities, troublesome pain conditions, or extensive muscle paralysis) to begin rehabilitation in a safer and less strenuous manner (14).

Recent fMRI studies have demonstrated that AOMI tasks activate brain regions associated with motor execution, emotional processing, and cognitive function, specifically the supplementary motor area, premotor cortex, primary motor cortex, cerebellum, posterior parietal regions, and the basal ganglia (15). Moreover, AOMI could promote neurotrophic factor expression, neuroplasticity, and the recruitment of alternative motor circuits (13). Growing evidence supports the beneficial effects of AOMI interventions on cognitive function and depression among the elderly with mobility impairment (16), post-total hip arthroplasty patients (17), adults with Parkinson’s disease (18), and those with vascular cognitive impairment (19). However, most studies remained at the pilot stage with a moderate to high risk of bias, while optimal intervention dosages have yet to be established. Furthermore, the movement/exercise components of imagery protocols vary across different populations. Based on theoretical foundations and practical evidence, we developed a home-based AOMI intervention specifically tailored for adults with SCI. The primary aim of this study is to assess the feasibility, acceptability, and preliminary effects of this pioneering intervention on cognitive function and depression in SCI. This study will specifically examine the intervention’s effects on attention and executive function, given that these cognitive domains are particularly susceptible to impairment in this population (20).

The second aim of this study is to evaluate the effects of AOMI on pain and self-efficacy for exercise. Preliminary evidence suggests that AOMI may reduce pain perception by sequentially activating motor cortical networks and increasing cortical excitability (21). This may have implications for cognitive impairment and depressive symptoms following SCI, as pain is a risk factor for both. In theory, AOMI could trigger positive changes in the self-efficacy for exercise, thereby facilitating increased physical activity engagement (22). Higher levels of physical activity are associated with a lower incidence of cognitive impairment and depression (23). Furthermore, multimodal MRI will be used to explore the potential neural mechanisms underlying AOMI. These insights can pave the way for updating current clinical practice and offer a deeper understanding of the neuroplasticity and brain’s functional reorganization among the SCI population post-intervention.

The home-based AOMI represents a promising intervention for improving cognitive function and alleviating depression in adults with SCI. However, the current evidence remains limited, underscoring the need for this research to (1) develop and validate population-specific AOMI intervention targeting cognitive function and depression; and (2) elucidate the underlying neurophysiological mechanisms underlying these therapeutic effects in SCI.

## 2 Methods

### 2.1 Study design

**Figure 1:**
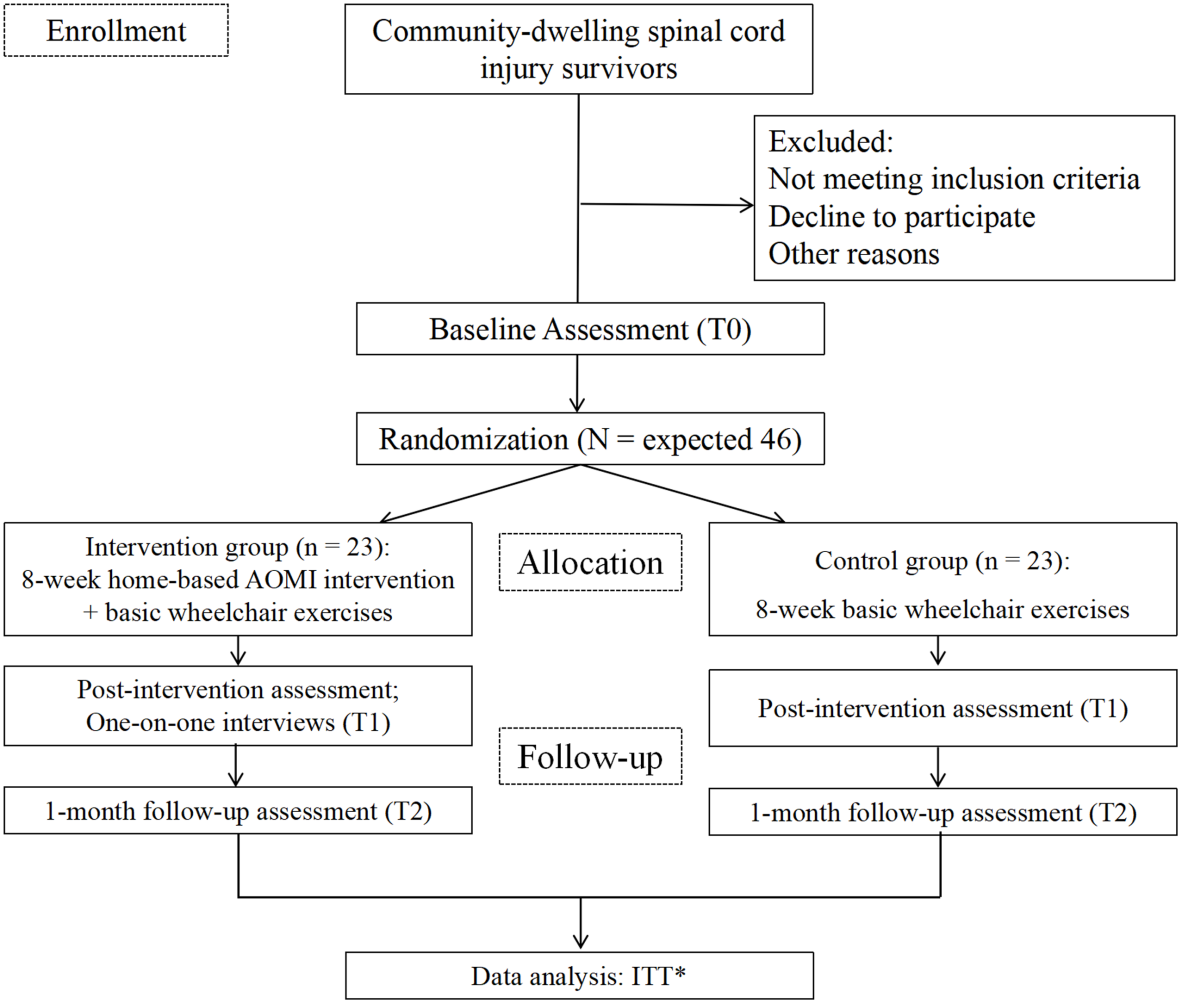
Study flow chart. *ITT, Intention-to-treat.

A prospective, assessor-blinded, two-arm pilot randomized controlled trial with repeated measures (pre-, post-intervention, and 1-month follow-up) will be conducted to evaluate the effectiveness of the AOMI intervention among adults with SCI. In total, 46 participants will be randomly assigned to either the intervention group, which will receive an 8-week AOMI intervention combined with basic wheelchair exercises, or the control group, which will watch landscape videos and perform basic wheelchair exercises. Participants will undergo MRI scans at baseline and after the intervention. One-on-one qualitative interviews will be implemented post-intervention to collect participants’ perceptions of effectiveness, intervention acceptability, strengths, limitations, and suggestions for further improvement. Participants’ recruitment began in March 2025. This study protocol complies with the Standard Protocol Items: Recommendations for Interventional Trials Statement (SPIRIT) (24) and the Consolidated Standard of Reporting Trials Statement (CONSORT): extension to randomized pilot and feasibility trials (25) (Figure 1). The protocol was prospectively registered at ClinicalTrials.gov (NCT06708026).

### 2.2 Participants

Participants are being recruited from community centers and non-governmental organizations in Hong Kong using advertisement posters. Eligibility screening for potential participants is conducted via online questionnaires and phone calls. Final enrollment is confirmed during an in-person visit, where participants receive a detailed overview of the study, provide informed consent, and complete a baseline assessment.

Severe cognitive impairment could impair participants’ motor imagery abilities, which may interfere with the assessment of intervention efficacy (26). Therefore, subjects will be included if they are (1) aged 18 years or above; (2) diagnosed with SCI for over 6 months, as per the International Standards for the Neurological Classification of SCI, confirmed by computed tomography or MRI; (3) with stable spinal systems and good vital signs, and residing in the community; (4) having no contraindications for MRI scans (e.g., absence of metal or electronic implants, non-pregnant status, and absence of claustrophobia); (5) having a mobile Internet terminal (usually a smartphone) and proficient independent or caregiver-assisted usage; and (6) able to communicate in Cantonese/Chinese and to provide written informed consent.

The exclusion criteria are as follows: (1) severe impairments in hearing, verbal communication, or vision; (2) participation in concurrent psychotherapy, physiotherapy, exercise, or relaxation interventions; (3) physically active for over 150 min of moderate-intensity exercise per week; (4) diagnosed mental disorders or substance misuse; and (5) severe cognitive impairment [indicated by the Hong Kong Montreal Cognitive Assessment (HK-MoCA) score ≤ 18] (27). No restrictions will be imposed regarding the level, cause, or completeness of the spinal cord injury.

### 2.3 Randomization and blinding

Randomization will be performed through the minimization method (28) to balance the pertinent influencing factors for cognitive function and the intervention effectiveness (i.e., motor imagery ability (29), age, and educational level). Minimization entails assigning intervention to succeeding trial participants based on the characteristics of previously enrolled participants (excluding the first participant who receives a random allocation) to enhance balance among the groups in key variables of interest. An independent administrative clerk will conduct this procedure by using the Minim software and will subsequently provide the researcher with a sequentially numbered, sealed, opaque envelope containing the group assignment for the participants. Due to the nature of the intervention, participants and the researchers who will enroll in the intervention process and qualitative interviews will not be blinded. However, the baseline and outcome assessor, as well as the data analyst, will remain blinded to group assignment.

### 2.4 Interventions

All participants will perform the same basic wheelchair exercises at home, following an instructional video. The video, which contains a set of low-intensity warm-up exercises, was developed by two experienced physiotherapists in Hong Kong (30). All participants will receive weekly phone calls to monitor progress and provide support during the training period. In order to avoid potential adverse events associated with the exercise (i.e., falls, joint pain, muscle strain, stress fracture, and exacerbation of existing injuries and disease symptomology) (31), the intensity of the workouts will be evaluated using the Borg ratings of perceived exertion (RPE), which should be maintained at less than 13 scores to ensure light to moderate intensity (32).

#### 2.4.1 Intervention group

Participants allocated to the AOMI group will receive, following the 10-min basic wheelchair exercise, a session of 20-min AOMI training. Based on the evidence from our scoping review, the intervention dosages are set as 3 sessions a week for a total of 8 weeks (Supplementary Table S1) (33). AOMI session duration of 20 min is widely adopted to minimize mental fatigue while maintaining the beneficial effects (34, 35). The AOMI training will be delivered through video recordings with verbal instructions that can be accessed via smartphones for streaming. After the randomization and prior to the intervention, participants will be informed in lay language about the concept of AOMI and its application in neurorehabilitation. Subsequently, they will engage in a face-to-face AOMI practice seminar guided by a trained researcher with motor imagery qualification. After the familiarization and demonstration, participants will receive the videos for AOMI. They will be required to watch the videos while simultaneously engaging in motor imagery of the same action at home.

The typical model, which is denoted by the acronym of AOMI components (i.e., PETTLEP, Physical, Environment, Task, Timing, Learning, Emotion, Perspective), was adopted for AOMI strategy development (36). These seven words represent a minimum checklist of AOMI components required to closely simulate real movements, aiming for optimal functional and physical equivalence. Based on the model and evidence from the scoping review (33), the home-based AOMI strategy specifically includes the following: asking participants to conduct AOMI while holding sport-specific implements, wearing comfortable clothing, and adopting the sitting position (Physical), and, where possible, practicing in a quiet home setting (Environment). The imagery is guided in synchrony with the real-time video with verbal instructions (Timing). To enhance familiarity with MI tasks, strategies such as gradual learning and repetition are employed (Learning). Motivational instructions combined with arousal-enhancing music are employed to facilitate the recall and recreation of exercise-associated affective states (Emotion). Furthermore, participants will be instructed and specified informed on the imagery viewpoint (Perspective). To maintain attention and enhance compliance (37), we designed three sets of wheelchair exercises and one set of walking activities as imagery contents (Task). These were presented in video format, based on the Canadian Spinal Cord Injury Physical Activity Guidelines (38) and our previous physical–psychological interventions specifically tailored for Hong Kong SCI populations (30). The imagery contents will change biweekly and become consistent every 2 weeks. Further details of the AOMI strategy and the specific tasks for motor imagery are provided in Supplementary Tables S2, S3, respectively.

#### 2.4.2 Control group

The control group will engage in a 10-min basic wheelchair exercise, followed by 20 min of watching landscape videos in their homes. The program will consist of three sessions per week over a total duration of 8 weeks.

### 2.5 Outcome measures

A trained research assistant, blinded to group allocation, will conduct a series of assessments at three time points: baseline (T0), after the 8-week intervention program (T1), and 1 month post-intervention (T2). Socio-demographic variables, feasibility and acceptability indexes, and effect outcomes are included. The effect outcomes include primary outcomes (global cognitive function and depression) and secondary outcomes (neurocognitive domains, multimodal MRI findings, chronic pain, self-efficacy for exercise, and motor imagery ability). The assessments will be conducted during participants’ visits to the study site (University campus) (Table 1).

**Table 1:** Data collection schedule.

Assessment items	Study period			
	Enrollment	Allocation	Post-allocation	
	−T1	T0 Baseline	T1 Post-intervention	T2 1-month follow-up
Enrollment				
Eligibility screening	X			
Informed consent	X			
Allocation		X		
Assessments				
Socio-demographics and medical history		X		
Feasibility of the intervention		X		X
Acceptability of the intervention			X	
Intervention effectiveness				
Primary outcomes				
Global cognitive function		X	X	X
Depression		X	X	X
Secondary outcomes				
Cognitive domain-attention		X	X	X
Cognitive domain-executive function		X	X	X
Multimodal MRI findings		X	X	
Chronic pain		X	X	X
Self-efficacy for exercise		X	X	X
Motor imagery ability		X	X	X

MRI, magnetic resonance imaging.

#### 2.5.1 Socio-demographics and injury characteristics

A self-designed form will gather participants’ socio-demographics and injury characteristics. This will encompass details including age, gender, marital status, occupation, exercise habits, educational level, mental medication, smoking and alcohol history, as well as information on the etiology, duration, neurological level of injury, complications, and American Spinal Injury Association (ASIA) Impairment Scale (AIS) category.

#### 2.5.2 Feasibility and acceptability

The feasibility indices include the recruitment rate, which is the percentage of participants who give consent after determining their eligibility; the retention rate, which refers to the number of participants who complete the study divided by all participants who agree to consent; and the dropout rate, which is the number of participants who drop out after randomization divided by all participants who agree to consent (39). The acceptability of the intervention will be indicated by (1) adverse event records related to the AOMI intervention; (2) intervention satisfaction, evaluated through the 8-item Client Satisfaction Questionnaire with a four-point Likert scale (40) and a qualitative interview. The one-on-one interviews will be conducted by a research assistant trained in qualitative research and possessing a background in psychology; (3) adherence rate, measured as the percentage of participants completing a minimum of 60% of interventions, equivalent to at least 15 sessions.

#### 2.5.3 Effectiveness of the home-based AOMI intervention

##### 2.5.3.1 Primary outcomes

The Chinese version of the Neuropsychiatry Unit Cognitive Assessment Tool (NUCOG) will be used to assess global cognitive function (41). This brief cognitive screening tool, specifically designed for neuropsychiatric patients (42, 43), has demonstrated clinical utility among individuals with SCI. The NUCOG consists of 21 items in 5 cognitive domains—attention, visuoconstructional ability, memory, executive function, and language—assigning scores from 0 to 20 for each area, resulting in a maximum total score of 100. Higher scores indicate higher levels of cognitive function. Due to the motor skills required for standard administration, survivors with SCI who have restricted or no hand function may face challenges. For those subjects, the adapted version of the NUCOG by Craig et al. will be utilized, a modification that does not affect the validity of NUCOG (44).

*Depression*: The level of depression will be evaluated using the second version of the Beck Depression Inventory (BDI-II), which consists of 21 items that assess two factors (cognitive and affective, and somatic symptoms) (45). The total possible score of BDI-II is 0 to 63; higher scores reflect higher levels of depression. The BDI-II has exhibited adequate reliability (Cronbach’s *α* coefficient of 0.94) when applied among individuals with SCI (46).

##### 2.5.3.2 Secondary outcomes

*Attention*: The Digit Span Test [Forward Span] and the oral version of the Symbol Digit Modalities Test (SDMT) will be used to assess attention. Referring to the former test, the examiner pronounces a list of digits at a rate of approximately one digit per second, and subjects are required to immediately repeat the list in the same order (47). Digit Span has high internal consistency, reliability, and construct validity (48). The SDMT involves participants verbally matching symbols with digits within a 90-s time frame (49). Higher numbers of correct responses indicate superior cognitive processing speed. The SDMT demonstrates good test–retest reliability and sensitivity to cognitive impairments across diverse populations (50).

*Executive function*: The Stroop Color Word Test and the Digit Span Tests [Backward Span] will be used. We will determine the former by subtracting the Color (congruent trials) from the Color-Word (incongruent trials) scores. It is a measure of response competition and interference with moderate-to-good reliability (51). Scoring is based on the time it takes to read (complete) incongruent trials relative to congruent trials. The backward digit span task uses the same procedure as the Digit Span forward task, except that in this case, subjects have to reproduce the sequence of digits in reverse order (52).

Multimodal MRI will be used to uncover possible mechanisms underlying the effectiveness of the AOMI intervention. The brain scanning session lasts around 40 min and includes the following sequences: T1-weighted anatomical scan for regional volume measurement, resting-state functional MRI (rs-fMRI) for assessing functional connectivity features and neural networks, task-based fMRI to indicate specific brain regions activated during AOMI tasks, and diffusion tensor imaging (DTI) to reflect the microstructural integrity of white matter tracts (53). During the rs-fMRI scan, subjects will be instructed to lie at rest with eyes open and fix on a bright cross-hair on a dark background. During the task-based fMRI, the subjects will be instructed to perform an “AOMI” fMRI task: subjects will be asked to imagine themselves performing the same exercise while watching the video representing wheelchair exercises. Videos will be presented from a dual perspective (first-person and third-person), and subjects will be asked to identify themselves and mentally simulate each situation (without moving the body). AOMI videos are alternated with resting periods in which subjects will be instructed to observe a static picture from the videos. The main acquisition parameters for each modality are listed in Supplementary Table S4. The multimodal MRI scans will be completed at the University Research Facility in Behavioral and Systems Neuroscience pre- and post-intervention on a 3-Tesla Siemens Prisma scanner, with a 32-channel head coil.

*Chronic pain*: The Chinese version of the Brief Pain Inventory (BPI-C) will be used to assess pain severity and interference (54). This questionnaire has been used in patients with SCI, with higher scores representing more violent pain (55).

*Self-efficacy for exercise*: The 9-item Chinese version of the self-efficacy for exercise (Cronbach’s *α* = 0.90) will be used to evaluate participants’ confidence level (from 0, not confident, to 10 very confident) regarding engaging in regular exercise (56). A higher score indicates greater exercise self-efficacy.

*Motor imagery ability*: The short version of the Kinesthetic and Visual Imagery Questionnaire (the KVIQ-10) will be used to evaluate the participants’ motor imagery ability (57). This questionnaire has 10 items, assessing both the visual and kinesthetic components of MI, and is particularly suitable for people with disabilities. The Chinese version of the KVIQ has been validated among stroke patients, showing good reliability and construct validity (49).

### 2.6 Sample size and sampling

The central limit theorem posits that a minimal sample size of 30–40 is enough for determining the mean, standard deviations, and 95% confidence intervals (58). This pilot RCT aims to assess the feasibility, acceptability, and preliminary effectiveness of the AOMI intervention, utilizing a generally acceptable sample size for preliminary analysis to evaluate intervention feasibility and estimate the between-group effect. Specifically, each study group will have 18 individuals to identify a standardized effect size of 0.40 with 80% power and a two-sided significance level of 5% (59). Taking into account a potential attrition rate of 20%, 23 participants per group (i.e., 46 participants in total) will be recruited by using convenience sampling. For one-on-one interviews, we will use a purposeful sampling of key informants, including those who completed at least 60% of the intervention sessions and those who did not, until we reach information saturation and find no relevant new codes in the data (60).

For MRI scans, a liberal threshold of *α* = 0.05 requires 24 participants (12 per group) to achieve 80% power at the single-voxel level for typical activations (61). To control error rates in multiple comparisons, doubling the number of participants is recommended to maintain this level of power. Due to the challenges of MRI data collection, each group will consist of a minimum of 12 participants undergoing pre- and post-intervention MRI scans. Notably, this sample size aligns with the median sample size observed in highly cited clinical fMRI studies published over the last three decades (62).

### 2.7 Data management and analysis

The data will be double-checked and entered into an Excel file with password protection for centralized management and stored on a dedicated research computer. During the data entry process, information will be de-identified and encrypted to protect the privacy of the participants. Only authorized personnel from our research team can access the data for analysis. An independent researcher not involved in the study implementation will be responsible for the regular monitoring of the data collection procedure.

#### 2.7.1 Quantitative data analyses

Quantitative data analyses will be conducted using IBM SPSS Statistics for Windows (Version 26.0. Armonk, NY: IBM Corp), with all statistical tests being two-sided and significance set at 0.05. The outcomes will be analyzed with an intention-to-treat approach (63). As for statistical description, the count data, such as gender will be presented as “frequency” and “percentage.” The continuous data that conform to the normal distribution (assessed by the W test coupled with a histogram) will be described using means ± standard deviation. As for group differences at baseline, post-intervention, and follow-up, *t-test* or Mann–Whitney *U* test will be performed for continuous variables; Pearson χ2 tests or Fisher exact test will be used for dichotomous variables. The generalized estimating equation (GEE) model, which could test the effects of group, time, and their interactions, will be used to analyze the effects of the intervention. Covariance (if any found in the baseline comparison) will be adjusted during the analysis of intervention effects. Cohen’s *d* will be used as the unit measure of effect size, and is interpreted as follows: 0.2 represents a small effect, 0.5 a moderate effect, and 0.8 a large effect. Cohen’s *d,* calculated by using the formula “difference in treatment and control group means divided by the pooled standard deviation,” is applicable for small sample sizes (64).

#### 2.7.2 Missing data management

The Little’s Missing Completely at Random test will be used to assess whether the missing values in a specific variable are unrelated to any other observed or unobserved variables (65). If the mechanism is missing at random, the multiple imputation will be conducted for handling the missing data due to its advantages of valid statistical inference even in the presence of a small sample size (66). For the GEE analysis, missing values are not imputed, as GEE can accommodate missing data and provides a natural way to deal with missing values (67).

#### 2.7.3 MRI data analysis

To acquire preliminary data on the association between the interventions and MRI metrics, per-protocol analyses will be conducted. Per-protocol analyses will include only those participants who complete at least 60% of the intervention sessions. Brain tissue total volume, gray matter, and white matter volumes, normalized for subject head size, will be estimated with SIENAX (part of FMRIB’s Software Library (FSL) 5.0.9). The tissue-type segmentation with partial volume estimation will be carried out to calculate the total volume of brain tissue (including separate estimates of volumes of gray matter and white matter). We will use Statistical Parametric Mapping 12 (SPM12) and the functional connectivity toolbox (CONN 20.b) in MATLAB 2021b for standard fMRI preprocessing and denoising procedures. Additionally, denoising will be achieved through linear regression to account for confounding effects. Finally, we will apply band-pass filtering [0.008, 0.1 Hz] to the blood-oxygen-level-dependent signals to focus on slow-frequency fluctuations while minimizing the influence of non-physiological, head-motion, and other noise sources (68).

To analyze fMRI data, differences of each group at baseline and post-intervention will be evaluated using the paired *t-test* in Statistical nonParametric Mapping (SnPM); differences between groups (intervention group vs. control group) will be assessed using a two-sample *t-test*. Differences between the two groups of the study over time will be evaluated using a General linear model, in which group and time will be included as distinct factors (2 × 2 factorial design). Multiple linear regression models will be used, with age, education level, and motor imagery ability as covariates, to assess the correlation between changes in MRI results and changes in cognitive function and depression data after intervention (69). For DTI data, statistical analyses will be performed using non-parametric permutation testing with 5,000 Monte Carlo simulations. We will look for differences in fractional anisotropy between the groups, accounting for age, educational level, and motor imagery ability as regressors of no interest in the general linear model. A two-sample *t* test will be used to evaluate the between-group differences in DTI metrices (e.g., path length, clustering coefficient, global efficiency, local efficiency, strength, and small world attribute). Association analysis between altered white matter microstructure imaging data and the assessments of cognitive function and depression will be analyzed with Spearman’s correlation coefficient (70).

#### 2.7.4 Qualitative data analysis

Content analysis will be conducted on qualitative data generated through one-on-one interviews. Research assistants will transcribe the recorded interviews and send them to the participants for verification. Two researchers with a background in qualitative data analysis will independently code the transcripts and cross-check them. Categories/subcategories and themes/subthemes will be gradually formed through repeated discussions in the research group (71).

### 2.8 Patient and public involvement

Patients and/or the public did not and will not engage in the design, execution, or reporting of this research. Participants will be invited to provide post-intervention evaluations. Qualitative interview findings will undergo participant validation, while both participants and clinicians will contribute to disseminating research outcomes within their professional networks (31).

### 2.9 Ethics and dissemination

This trial follows the Declaration of Helsinki and has been approved by the Institutional Review Board of the Hong Kong Polytechnic University (HSEARS20240716007-01). All eligible subjects will be asked to participate after receiving an explanation of the aim of the study, the potential benefits and risks, confidentiality and autonomy issues, and the right to withdraw at any time. Eligible subjects who agree to participate will be asked to sign a consent form before the baseline assessments of the study (Supplementary material, consent form). In addition, the consent forms will also be obtained from participants who will accept MRI scans and one-on-one interviews. If any safety concerns emerge in the process, the participants will be evaluated and managed by our research team (we have a physiotherapist and nurses in our team). If participants experience severe dizziness, fatigue, pain, injury, or illness during the intervention, they can immediately withdraw from the study and be referred to healthcare professionals for further assessment and care. The study results will be disseminated in peer-reviewed journals and conference presentations. The researcher will report only aggregate findings, not individual data, to the public.

## 3 Discussion

This study focuses on cognitive impairment and depression, both of which are significant issues in adults with SCI that have not yet been well addressed. Although physical exercise-based interventions have shown preliminary efficacy for these two conditions in SCI, their clinical application remains limited to individuals with certain levels of motor function (72). Conversely, AOMI exhibits comparable therapeutic benefits while accommodating a broader SCI population, including those with severe motor impairments (16, 18). Furthermore, AOMI can be self-administered following proper training. Emerging evidence supports the positive effects of AOMI on other disabled cohorts. This study will be the first to specifically develop and explore the therapeutic potential of a home-based AOMI intervention for improving cognitive function and depression following SCI. Additionally, it will further evaluate the efficacy of improving attention and executive function, two cognitive domains particularly vulnerable in individuals with SCI and crucially associated with successful community reintegration (20).

In recent years, few studies have implemented AOMI for SCI survivors targeting pain relief (73). While its feasibility has reached consensus, the therapeutic effects remain controversial. Given that pain represents a risk factor for both primary outcomes, this study will additionally examine whether AOMI might indirectly enhance cognitive function and reduce depression in adults with SCI through pain modulation. Following SCI, individuals frequently experience diminished exercise self-efficacy, leading to lower physical activity levels and subsequent negative impacts on both cognitive function and mood (74). While prior research has established AOMI’s positive effects on exercise self-efficacy in healthy populations and athletes (75), this study specifically evaluates its efficacy in adults with SCI. If these positive effects are preliminarily demonstrated, this home-based AOMI could offer a non-invasive, accessible, cost-efficient new path to the healthcare plans of SCI rehabilitation.

This pilot RCT adopts a multidisciplinary approach, incorporating psychological, neurological, and neuropsychological assessments. The utilization of multimodal MRI enables a better understanding of the neural mechanism behind the AOMI and post-SCI neuroplastic changes. These findings will establish an empirical foundation for developing mechanism-driven interventions targeting cognitive impairment and depression in adults with SCI.

This study has several limitations that should be acknowledged. First, this pilot RCT can only show the preliminary effectiveness of the home-based AOMI intervention. Furthermore, the small sample size and potential selection bias resulting from convenience sampling may limit the generalizability of the findings. However, the results of this pilot study will provide valuable insights for designing a future full-scale RCT, which could generate much-needed level one evidence regarding the effectiveness of AOMI. Second, due to the inherent nature of the intervention, neither the interventionists nor the participants could be effectively blinded. Although we will implement weekly phone monitoring of intervention adherence and design an active control condition, potential performance bias may remain a limitation. Third, a defining characteristic of AOMI is the absence of overt physical actions during the imagery process. This inherent feature makes objective performance evaluation challenging, as AOMI relies on mental movement representation. We will employ qualitative methods (i.e., training diaries and post-intervention interviews) to assess whether participants really engaged in AOMI and whether they truly imagined the intended content. However, integrating objective methods such as neuroimaging techniques would better evaluate intervention fidelity. Due to resource constraints, we will consider this monitoring method in future research.
