# Supplementary material for: Effects and mechanisms of a home-based action observation and motor imagery intervention on cognitive function and depression in spinal cord injury: a pilot randomized controlled trial protocol
Source: Front Neurol. 2025 May 16;16:1578323. doi: 10.3389/fneur.2025.1578323 (PMC12124480; doi:10.3389/fneur.2025.1578323)
Supplement: Supplementary file 1 [file Data_Sheet_1.docx]

***Supplementary File***

**Table S1** Summative results of intervention delivery dosages: evidence from the scoping review

| **Category** | **Number of studies** | **Percentage** | **Studies** |
| --- | --- | --- | --- |
| **Duration*** |  |  |  |
| ＜ 1 month | 3 | 13.6% | [1-3] |
| 1 month | 5 | 22.7% | [4-8] |
| 1.5 months | 4 | 18.2% | [9-12] |
| 2 months | 6 | 27.3% | [13-18] |
| 3 months | 4 | 18.2% | [19-22] |
| **Frequency** |  |  |  |
| 2 times per week | 4 | 18.2% | [14, 20-22] |
| 3 times per week | 4 | 18.2% | [1, 9, 12, 17] |
| 4~6 times per week | 4 | 18.2% | [7, 10, 16, 19] |
| Daily | 10 | 45.4% | [2-6, 8, 11, 13, 15, 18] |
| **Length of each session** |  |  |  |
| 15~＜20 mins | 5 | 22.7% | [3, 6, 7, 13, 15] |
| 20-30 mins | 9 | 40.9% | [2, 5, 8, 9, 14, 16-18, 22] |
| ＞30 mins | 6 | 27.3% | [1, 10, 12, 19-21] |
| Not report | 2 | 9.1% | [4, 11] |

*The duration of interventions varied among studies and was related to the frequency: daily administration was common for interventions lasting one month or less, and 2-3 times per week for those lasting around 2 months.

MI: motor imagery.

**Table S2** The elements of the AOMI strategy based on the PETTLEP model and evidence

| **Elements** | **Rationale** | **Detailed description and evidence** |
| --- | --- | --- |
| **P** (Physical) | The physical state of practitioners during MI. To closely resemble the actual motor preparation and execution, the dressing, position, and sport-specific implements will be considered. | Practice at any time of the day when alert, except at night.   - **Dressing:** relaxed clothing ^[23]^. - **Position:** Sit in an upright body position; relax shoulders ^[7, 9, 14]^. - **Exercise equipment:** hold a resistance band or water bottles (depending on the specific task) ^[24]^. |
| **E** (Environment) | The place where the MI is performed. As this is a home-based intervention, the virtual environment will be created to simulate the sensory experience of actual exercise as closely as possible. | - **Real environment:** a quiet and safe place at home; - **Virtual environment:** a gym or park, created through the visual and auditory cues of the videos ^[6, 9, 14, 22]^. |
| **T** (Tasks) | The imagery contents. Based on the evidence from the scoping review, MI tasks aimed at improving cognitive function and depression should include functional exercises targeted at functional deficits and walking. | - **Weeks 1-6:** Wheelchair fitness exercises specifically developed for SCI survivors (the detailed description of tasks can be found in **Table S3**) ^[12, 14, 20]^. The imaginary contents will change biweekly and become consistent every two weeks. - **Weeks 7-8:** Both indoor and outdoor walking, with a detailed task description available in **Table S3** ^[7-9, 12, 14, 17, 19, 20]^. |
| **T** (Timing) | The pace at which the MI is completed. | The **visual and auditory cues** from the videos will be used to indicate temporal rhythm (e.g., watch the video, follow the model, and have 10 seconds to repeat imagining this action 3 times) ^[10]^. |
| **L** (Learning) | Strategies will be conducted to help participants become familiar, as gradual learning and repetition enhance memory of the motor tasks. | - **Familiar:** Each MI task will be demonstrated through the researcher (when participants come to campus for baseline assessment) and videos ^[7, 9, 19]^. - **Gradual learning:** see familiarisation. Exercise tasks will progress as participants gained proficiency in the imagined movements. The weekly phone call will provide guidance and necessary adjustments. - **Repetition:** Each task will be repeated several times to consolidate motor memory and promote the creation of more vivid MI. |
| **E** (Emotion) | Motivational and arousal-enhancing instructions and photos of positive exercise endings will be provided in videos to promote the mental recreation of emotions felt during actual exercise. | - Examples of motivational and arousal-enhancing instructions: “Punching it out. This is your last one, guys. Keep it up. Try to get that heart rate as high as you can. Good job! Give me all the punches you can. Speed it up, speed it up. You get it!” ^[7, 9]^ - Photos of positive exercise endings: At the final stage of each video, provide photos of positive exercise endings, guiding participants to imagine the pleasant scenes of successful execution ^[24]^. |
| **P** (Perspective) | The viewpoint of the participant during imagery. | Request the use of the internal/first-person (through the participant's eyes) perspective ^[7, 10, 15, 17]^. |

AOMI: action observation and motor imagery; PETTLEP: Physical, Environment, Task, Timing, Learning, Emotion, Perspective; SCI: spinal cord injury.

# **Table S3** Specific tasks for AOMI

| **Stages of the program** | **Rationale** | **Exercise description** |
| --- | --- | --- |
| **Stage 1**  (Weeks 1 and 2) | Warm-up session | - Running arm swing - Breaststroke arm swing (upper, middle, lower) - [Freestyle swimming arm propulsion](https://www.youtube.com/watch?v=6phU3vN8ApI) - Neck flexion - Neck rotation - Lateral neck flexion - Shoulder rolls forwards and backwards - Arm flexion and extension - Forearm rotation - Seated body rotation |
|  | Core exercise session  (cardio exercises) | - Arm circles (forwards and backwards) - Scapular push-up exercise - Resistance exercises for biceps - Running arm swing - Virtual rope climb exercise - Straight punch - Hook punch - Uppercut - Boxing speedball |
|  | Cool-down session | - Neck flexion and rotation - Upper back stretch - Forearm (extensor) stretch - Forearm (flexor) stretch - Seated twist stretch |
| **Stage 2**  (Weeks 3 and 4) | Warm-up session | - Running arm swing - Breaststroke arm swing (upper, middle, lower) - [Freestyle swimming arm propulsion](https://www.youtube.com/watch?v=6phU3vN8ApI) - Neck flexion - Neck rotation - Lateral neck flexion - Shoulder rolls forwards and backwards - Arm flexion and extension - Forearm rotation - Seated body rotation |
|  | Core exercise session  (strength training by using two water bottles) | - Water bottle overhead press - Water bottle bicep curls - Water bottle arm raises - Lateral Shoulder Raise-water bottle - Water bottle shoulder retractions - Water bottle chest squeeze - Water bottle tricep kickbacks |
|  | Cool-down session | - Neck flexion and rotation - Upper back stretch - Forearm (extensor) stretch - Forearm (flexor) stretch - Seated twist stretch |
| **Stage 3**  (Weeks 5 and 6) | Warm-up session | - Running arm swing - Breaststroke arm swing (upper, middle, lower) - [Freestyle swimming arm propulsion](https://www.youtube.com/watch?v=6phU3vN8ApI) - Neck flexion - Neck rotation - Lateral neck flexion - Shoulder rolls forwards and backwards - Arm flexion and extension - Forearm rotation - Seated body rotation |
|  | Core exercise session  (resistance training by using resistance bands) | - Bicep curls - Reverse fly - Chest fly - Crunches - Cross crunches - Squats with a lat pull - Side steps with single arm rows |
|  | Cool-down session | - Neck flexion and rotation - Upper back stretch - Forearm (extensor) stretch - Forearm (flexor) stretch - Seated twist stretch |
| **Stage 4**  (Weeks 7 and 8) | Walking | - Walking in place - Walking straight - Walking straight and turning 180° - Sit-to-stand and walking straight - Sit-to-stand, walking straight and turning 180° - Tandem walking (Alternately move both feet along a straight line, with one heel closely following the toes of the other foot as you walk forward) - Walking backward - Climbing stairs - Climbing mountains (walking surface, speed, and slope steepness will change with the improvement of familiarity) |

**Table S4.** Multi-model MRI protocol

| **Modality** | **Duration (min)** | **Voxel, Field of view** | **Key parameters** |
| --- | --- | --- | --- |
| T1-weighted MRI | 6:54 | 0.8*0.8*0.8 mm^3^  256*256 mm^2^ | Slices per Slab = 208  TE = 2.22 ms  TI/TR = 1120/2500 ms |
| Resting-state fMRI | 8:14 | 3.0*3.0*3.0 mm^3^  192*192 mm^2^ | Slices = 38  TE/TR = 32/2000 ms  Acceleration Factor PE = 4 |
| Task-based fMRI | 7:20 | 3.0*3.0*3.5 mm^3^  240×240 mm^2^ | TE = 30 ms  TR = 1000 ms  flip angle = 58°  34 interleaved axial slices  inter-slice gap = 10% of the slice thickness  multiband acceleration factor = 2  GRE field maps (short TE = 4.92 ms, long TE = 7.38 ms) for geometric distortion correction of EPI images.  Subjects will perform a “AOMI” fMRI task: subjects will be asked to imagine themselves performing the same exercise while watching the video representing wheelchair exercises. Videos will be presented using E-prime 3.0 and subjects will be asked to identify themselves and mentally simulate each situation (without moving the body). A block design (ABAB) will be used, where the activation A (lasting about 20 seconds) corresponds to the mental imagination of the action while watching the video, and the resting period B (lasting about 16 seconds) requires subjects to observe a static screenshot image from the video. Each period will be repeated 12 times. |
| DTI | 5:48 + 5.48  (AP + PA) | 1.5*1.5*1.5 mm^3^  210*210 mm^2^ | Slices = 92  TE/TR = 89/3200 ms  b-value 1 = 0 s/mm^2^  b-value 2 = 1500 s/mm^2^  Diff. Directions = 99 |

AP, Antero-Posterior; DTI, Diffusion Tensor Imaging; EPI, Echo Planar Imaging; fMRI, functional magnetic resonance imaging; PA, Postero-Anterior; PF, partial Fourier; MRI, magnetic resonance imaging; rs-fMRI, resting-state functional magnetic resonance imaging; TE, echo time; TI, Inversion time; TR, repetition time.

**References**

[1] Haire CM, Vuong V, Tremblay L, *et al*. Effects of therapeutic instrumental music performance and motor imagery on chronic post-stroke cognition and affect: A randomized controlled trial. *NeuroRehabilitation.* (2021) 48:195-208. doi:10.3233/NRE-208014

[2] Jiang H, Chen C, Hao X. The intervention effects of motor-guided imagery training on anxiety, depression, and quality of life in stroke patients. *Chinese Journal of Rehabilitation Medicine.* (2020) 35:738-740.

[3] Wu Y, Fei X, Gu Y, *et al*. Influence of combining motor imagery therapy with counterbalance exercise on fall efficacy of the elderly. *Chinese Journal of Modern Nursing.* (2016) 22:3364-3367.

[4] Dijkerman HC, Ietswaart M, Johnston M, *et al*. Does motor imagery training improve hand function in chronic stroke patients? A pilot study. *Clin Rehabil.* (2004) 18:538-549. doi:10.1191/0269215504cr769oa

[5] Hu Q. Effect of motor imagery and cognitive function training on BI, FCA scores and quality of life in elderly patients with cerebral infarction. *Medical Journal of the Chinese People's Armed Police Forces.* (2018) 29:607-610.

[6] Luo M, Zhou H, Hao X, *et al*. Effect of characteristic guided motor imagery on anxiety,depression,and disability acceptance in stroke patients with hemiplegia. *Journal of HeBei United University (Health Sciences).* (2022) 24:137-142.

[7] Seebacher B, Kuisma R, Glynn A, *et al*. Effects and mechanisms of differently cued and non-cued motor imagery in people with multiple sclerosis: A randomised controlled trial. *Mult Scler. (*2019) 25:1593-1604. doi:10.1177/1352458518795332

[8] Zhang N, Cai H, Han X, *et al*. Effects of exercise guided imagination training combined with duloxetine on neurocognitive function,se-rum NGF and BDNF levels in patients with depression after cerebral infarction. *Journal of International Psychiatry.* (2022) 49:672-674, 681.

[9] Kim S, Cho S. Virtual Reality Programs and Motor Imagery Training Reduce Plantar Pressure and Depression in Isolated Older Adults. *International Journal of Gerontology.* (2022);16.

[10] Mahmoud LSE, Abu Shady NAE, Hafez ES. Motor imagery training with augmented cues of motor learning on cognitive functions in patients with Parkinsonism. *International Journal of Therapy and Rehabilitation.* (2018) 25:13-19.

[11] Salik SY, Kaya N, Yalcinkaya G, *et al*. The effects of the addition of motor imagery to home exercises on pain, disability and psychosocial parameters in patients undergoing lumbar spinal surgery: A randomized controlled trial. *Explore (NY).* (2021)17:334-339. doi:10.1016/j.explore.2020.02.001

[12] Sarasso E, Agosta F, Piramide N, *et al*. Action Observation and Motor Imagery Improve Dual Task in Parkinson's Disease: A Clinical/fMRI Study. *Mov Disord.* (2021) 36:2569-2582. doi:10.1002/mds.28717

[13] Gong W. Effect of motor imagery therapy on cognitive function of patients with stroke. *Chinese Journal of Contemporary Neurology and Neurosurgery.* (2017) 17:415-420.

[14] Kahraman T, Savci S, Ozdogar AT, *et al*. Physical, cognitive and psychosocial effects of telerehabilitation-based motor imagery training in people with multiple sclerosis: A randomized controlled pilot trial. *J Telemed Telecare.* (2020) 26:251-260. doi:10.1177/1357633X18822355

[15] Liu L, Li X, Sun J. The application of remote motor imagery therapy in home-based rehabilitation care for stroke patients. *International Journal of Nursing.* (2020) 39:3821-3824.

[16] Liu W, Li Z, Xie Y, *et al*. Effects of a Combined Motor Imagery and Action Observation Intervention on Vascular Cognitive Impairment: A Randomized Pilot Study. *Am J Phys Med Rehabil.* (2022) 101:358-366. doi:10.1097/PHM.0000000000001827

[17] Marusic U, Grospretre S, Paravlic A, *et al.* Motor Imagery during Action Observation of Locomotor Tasks Improves Rehabilitation Outcome in Older Adults after Total Hip Arthroplasty. *Neural Plast.* (2018) 2018:5651391. doi:10.1155/2018/5651391

[18] Wang Q. The impact of combined motor imagery training and cognitive training on neuroplasticity in patients with ischemic stroke. *Electronic Journal of Practical Clinical Nursing Science.* (2019) 4:101-102, 104.

[19] Chen H. The impact of combined motor imagery therapy and cognitive intervention on patients with postoperative aneurysmal subarachnoid hemorrhage. *Chinese General Practice Nursing.* (2022) 2:370-372.

[20] Paolucci T, Cardarola A, Colonnelli P, et al. Give me a kiss! An integrative rehabilitative training program with motor imagery and mirror therapy for recovery of facial palsy. *Eur J Phys Rehabil Med.* (2020) 56:58-67. doi:10.23736/S1973-9087.19.05757-5

[21] Tamir R, Dickstein R, Huberman M. Integration of motor imagery and physical practice in group treatment applied to subjects with Parkinson's disease. *Neurorehabil Neural Repair.* (2007) 21:68-75. doi:10.1177/1545968306292608

[22] Zhang Q, Huang Y. The impact of remote motor imagery therapy combined with Barthel Index-based hierarchical home care on stroke patients. *Chinese General Practice Nursing.* (2023) 21:4388-4392.

[23] Wakefield C, Smith D. Perfecting practice: Applying the PETTLEP model of motor imagery. *Journal of Sport Psychology in Action*. (2012) 3:1-11. [doi:10.1080/21520704.2011.639853](https://doi.org/10.1080/21520704.2011.639853)

[24] Morone G, Ghanbari Ghooshchy S, Pulcini C, *et al*. Motor imagery and sport performance: A systematic review on the PETTLEP model. *Applied Sciences.* (2022) 12:9753. doi:10.3390/app12199753

Informed Consent Form

**Development and Evaluation of a Home-based Action Observation and Motor Imagery Intervention on Cognitive Function and Depression among Adults with Spinal Cord Injury: A Pilot Randomized Controlled Trial**

I, ___________________, hereby agree to participate in the above-mentioned study conducted by Dr. Yan Li from the School of Nursing, The Hong Kong Polytechnic University.

I understand that the data collected from this study may be used for future research and publication purposes; however, my privacy rights will be protected, and my personal information will not be disclosed.

The researchers have provided a clear explanation of the research procedures outlined in the attached information sheet, and I understand the potential benefits and risks involved. I am voluntarily participating in this research project.

I acknowledge that I have the right to raise any questions regarding any aspect of the study and have the right to withdraw at any time without facing any penalty.

Participant's Name: ______________________________________

Participant's Signature: ______________________________________

Parent or Guardian (if applicable) Name: _________________________

Parent or Guardian (if applicable) Signature: _________________________

Researcher’s Name: _____________________________________

Researcher’s Signature: _____________________________________

Date: _____________________________________________
